# Characteristic Conformation of Mosher’s Amide Elucidated Using the Cambridge Structural Database

**DOI:** 10.3390/molecules200712880

**Published:** 2015-07-16

**Authors:** Akio Ichikawa, Hiroshi Ono, Yuji Mikata

**Affiliations:** 1 Division of Insect Sciences, National Institute of Agrobiological Sciences, Tsukuba, Ibaraki 305-8634, Japan; 2 Analytical Science Division, National Food Research Institute, Tsukuba, Ibaraki 305-8642, Japan; E-Mail: ono@affrc.go.jp; 3 Department of Chemistry, Biology, and Environmental Science, Faculty of Science, Nara Women’s University, Kita-Uoya Nishi-machi, Nara 630-8506, Japan; E-Mail: mikata@cc.nara-wu.ac.jp

**Keywords:** chiral recognition, chirality, crystal engineering, Mosher’s method, MTPA

## Abstract

Conformations of the crystalline 3,3,3-trifluoro-2-methoxy-2-phenylpropanamide derivatives (MTPA amides) deposited in the Cambridge Structural Database (CSD) were examined statistically as *R*_acid_-enantiomers. The majority of dihedral angles (48/58, *ca.* 83%) of the amide carbonyl groups and the trifluoromethyl groups ranged from –30° to 0° with an average angle *θ*^1^ of −13°. The other conformational properties were also clarified: (1) one of the fluorine atoms was antiperiplanar (*ap*) to the amide carbonyl group, forming a staggered conformation; (2) the MTPA amides prepared from primary amines showed a *Z* form in amide moieties; (3) in the case of the MTPA amide prepared from a primary amine possessing secondary alkyl groups (*i.e.*, Mosher-type MTPA amide), the dihedral angles between the methine groups and the carbonyl groups were *syn* and indicative of a moderate conformational flexibility; (4) the phenyl plane was inclined from the O–C_chiral_ bond of the methoxy moiety with an average dihedral angle *θ*^2^ of +21°; (5) the methyl group of the methoxy moiety was *ap* to the *ipso*-carbon atom of the phenyl group.

## 1. Introduction

**Figure 1:**
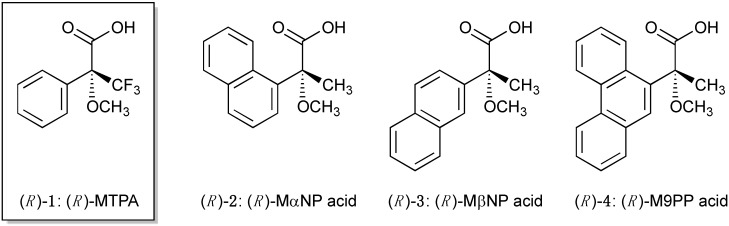
Structures of chiral resolving agents.

**Figure 2:**
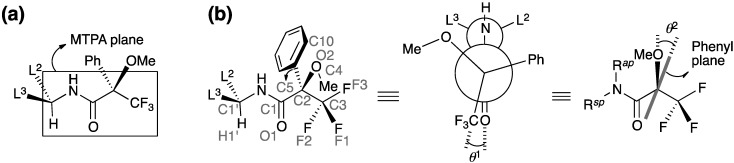
Major conformation of (*R*_acid_)-MTPA amide proposed in (**a**) previous studies and (**b**) this study. The Newman-like projection originally proposed by Mosher *et al*. [3] was modified in (**b**). Three covalent bonds separate the two chiral centers C1′ and C2; therefore, L^3^–C1′–L^2^ moiety is more flat in this projection. The MTPA ester exhibited the equivalent conformation with substitution of NH for O [9].

NMR using chiral resolving agents is a powerful technique, along with X-ray crystallography and circular dichroism, for assignment of the absolute configuration of organic compounds [1]. Mosher *et al*. developed 3,3,3-trifluoro-2-methoxy-2-phenylpropanoic acid (MTPA, **1**, Figure 1) and constructed the conformational model of the MTPA amide and the MTPA ester derived from a primary amine and a secondary alcohol, respectively (Figure 2a) [2,3,4]. Considering the shielding effect of the phenyl ring, the relative stereochemistry of the MTPA amide and the MTPA ester could be elucidated based on a mutual comparison of the ^1^H-NMR chemical shifts of their diastereomers; namely, upfield shifts are observed in substituent L^2^. Therefore, the absolute configuration of the amine moieties and the alcohol moieties could be clarified using stereochemistry of the MTPA moiety as an internal standard. Kusumi *et al*. modified this method using two-dimensional NMR spectroscopy [5,6,7,8].

We studied on three chiral resolving agents [*i.e.*, MαNP acid (**2**), MβNP acid (**3**), and M9PP acid (**4**)] based on their enantioresolution of chiral alcohols and elucidation of absolute configurations [10,11,12,13,14,15,16,17,18,19,20,21,22,23,24]. The resolving ability of **2** is superior to that of **1** in normal phase HPLC. The diastereomeric MαNP esters also exhibit large chemical shift differences (Δδ values) in ^1^H-NMR spectroscopy [12,18].

In 2013, we elucidated the crystal structure of Mosher’s salt prepared from (*R*)-**1** and (*R*)-1-phenylethylamine using X-ray crystallography [9]; Mosher *et al*. prepared this compound via the enantioresolution of *rac*-**1** in ethanol as a less soluble salt [2]. In the course of this study, we found that a number of crystal structures of MTPA amides and MTPA esters were deposited in the CSD.

Each molecular structure is influenced by packing force in crystal [25]. However, statistical analyses of the crystal structures elucidated the relative stability of each conformer [25]. Therefore, we reported statistical analyses of the crystal structures of MTPA esters [9].

The properties of the major conformation of the crystalline MTPA ester are as follows [9]: (1) the ester carbonyl group is synperiplanar (*sp*, dihedral angle 0° to ±30°) [26] to the trifluoromethyl group; (2) the trifluoromethyl group is in the staggered conformation; (3) the methine group of the alcohol moiety is *syn* to the carbonyl group; (4) the phenyl plane is inclined from the O–C_chiral_ bond of the methoxy moiety; (5) the methyl group of the methoxy moiety is antiperiplanar (*ap*, dihedral angle ±150° to 180°) [26] to the *ipso*‑carbon atom of the phenyl group. Thus, our database study proposed a modified conformational model of the MTPA ester.

In this report, we perform the statistical analyses of the crystal conformations of MTPA amides deposited in the CSD [27,28,29,30,31,32,33,34,35,36,37,38,39,40,41,42,43,44,45,46,47,48,49,50,51,52,53,54,55,56,57,58,59,60,61,62,63,64,65,66]. The conformational data of the crystalline MTPA amide moiety (a total of 58), which had been prepared from: (i) primary amines; (ii) secondary amines; (iii) aniline derivatives [tetrakis-MTPA amides of ruthenium(II) porphyrin complexes]; (iv) diethyl 1-aminoalkylphosphonate derivatives; (v) benzotriazole; (vi) an oxazolidine-2-selone derivative; (vii) a thiocarbamide derivative; and (viii) a *p*-toluenesulfonamide derivative, were statistically analyzed as *R*_acid_-enantiomers.

Conformational features of the MTPA amide moiety were similar to those of the MTPA esters, with less diversity (Figure 2b). The features of the major conformation of crystalline MTPA amides are as follows: (1) the amide carbonyl group is *sp* to the trifluoromethyl group with an average dihedral angle *θ*^1^ of −13°; (2) the trifluoromethyl group is in the staggered conformation; (3) the amide moiety of the secondary MTPA amide is in the *Z* form (*i.e.*, R*^ap^* = H, R*^sp^* = alkyl group); (4) H1′ of the amine moiety is *syn* to the carbonyl carbon atom C1; (5) the phenyl plane is inclined from the O2–C2 bond with an average dihedral angle *θ*^2^ of +21°; (6) the methyl group of the methoxy moiety is *ap* to C5 of the phenyl group.

Structural elucidation of chiral amines is important, because a considerable number of biologically active natural products and pharmaceuticals contain key chiral amine moieties [67,68,69]. The number of entries in the CSD is increasing rapidly; therefore, the crystal database is important for structural chemistry. The statistical analyses of crystal data have increased our understanding on the properties of acid **1** and have established valuable insights for Mosher’s method and crystal engineering [70,71] of MTPA derivatives.

## 2. Results and Discussion

### 2.1. Crystal Structures of MTPA Amides Deposited in the CSD

The crystal structures of the MTPA amide moieties were searched in the CSD using ConQuest software. Table 1 shows the original dihedral angles of (*R*_acid_)- and (*S*_acid_)-MTPA amides [27,28,29,30,31,32,33,34,35,36,37,38,39,40,41,42,43,44,45,46,47,48,49,50,51,52,53,54,55,56,57,58,59,60,61,62,63,64].

**Table 1:** Dihedral angles of crystalline MTPA amides ^(a)^.

No.	CCDC Number ^(b)^	Reference	Chirality of MTPA ^(a)^	Amine Moiety	R *^sp^*	R *^ap^*	O1–C1–C2–C3 (*θ*^1^)	C1–C2–C3–F3	C1'–N–C1–O1	X1′′–N–C1–O1 ^(c)^	H1′–C1′–N–C1	O2–C2–C5–C10 (*θ*^2^)	C1–C2–O2–C4
1 ^(d)^	199868	[27]	*R*	Primary amine	Secondary alkyl group	H	−13.1(2)	−172.0(1)	7.1(2)	−172.9	−47.4	23.3(2)	54.2(2)
2 ^(d)^	199868	[27]	*R*	Primary amine	Secondary alkyl group	H	−7.3(2)	−174.6(1)	6.3(2)	−173.7	−51.5	29.4(2)	50.4(2)
3 ^(d)^	222942	[28]	*R*	Primary amine	Secondary alkyl group	H	30.3(3) ^(e)^	175.6(2)	−4.2(5)	169(4)	47.0	11.2(7)	−44.4(4)
4 ^(d)^	603055	[29]	*S*	Primary amine	Secondary alkyl group	H	35.0(4) ^(e)^	165.9(3)	0.5(5)	−179.5	15.2	−43.7(4)	−72.0(3)
5	651954	[30]	*R*	Primary amine	Primary alkyl group	H	−26(2)	−168(1)	−6(2)	173	–	24(2)	72(1)
6	651954	[30]	*R*	Primary amine	Primary alkyl group	H	−25(2)	−166(1)	8(2)	−172	–	22(2)	60(2)
7 ^(d)^	678252	[31]	*R*	Primary amine	Secondary alkyl group	H	−22.6(4)	−171.7(3)	9.5(5)	−170.6	29.6	19.7(4)	58.4(3)
8 ^(d)^	678252	[31]	*R*	Primary amine	Secondary alkyl group	H	−14.4(5)	−173.2(3)	5.2(6)	−174.8	28.3	22.5(4)	54.2(4)
9 ^(d,f)^	703912	[32]	*R*	Primary amine	Secondary alkyl group	H	−67.7(6) ^(e)^	−174.6(5)	3.7(9)	170(5)	−22.5	−58.9(7)	−162.7(5)
10	734247	[33]	*R*	Primary amine	Primary alkyl group	H	−29.1(5)	−168.3(3)	−2.9(6)	177.0	–	24.2(5)	62.9(4)
11	739753	[34]	*R*	Primary amine	Primary alkyl group	H	−36.0(2) ^(e)^	−164.7(2)	7.1(3)	−172.9	–	49.8(2)	64.5(2)
12 ^(d)^	1218697	[35]	*R*	Primary amine	Secondary alkyl group	H	−9.8(3)	−176.9(2)	−3.0(4)	171(2)	1.3	12.0(3)	55.5(3)
13 ^(d)^	1229820	[36]	*R*	Primary amine	Secondary alkyl group	H	−31(1) ^(e)^	−169.2(8)	15(2)	136	−7	21	64
14 ^(d)^	1277744	[37]	*R*	Primary amine	Secondary alkyl group	H	−28.4(3)	−170.3(2)	3.9(4)	−176.0	−14.4	26.9(3)	65.2(3)
15 ^(g)^	140352	[38]	*R*	Secondary amine	Secondary alkyl group	Primary Alkyl group	−4.7(5)	−175.4(3)	5.3(6)	−174.7(4)	20.4(6)	27.5(5)	53.4(4)
16 ^(g)^	167289	[39]	*S*	Secondary amine	Secondary alkyl group	Primary alkyl group	5.2(2)	177.7(1)	−0.5(2)	−177.4(2)	10(2)	−11.7(2)	−45.0(2)
17 ^(g)^	241708	[40]	*S*	Secondary amine	Secondary alkyl group	Primary alkyl group	6.9(2)	176.0(1)	−1.7(2)	169.5(1)	48.1	−15.4(2)	−45.1(2)
18 ^(g)^	251663	[41]	*R*	Secondary amine	Secondary alkyl group	Primary alkyl group	−12.4(2)	−172.3(1)	1.8(2)	−168.2(1)	24.3	24.5(2)	56.7(2)
19	288331	[42]	*S*	Secondary amine	Secondary alkyl group	Secondary alkyl group	7.7(3)	176.5(2)	8.7(3)	174.0(2)	−1.3	−14.0(3)	−50.1(3)
20	296547	[43]	*R*	Secondary amine	Primary alkyl group	Primary alkyl group	–13.4(8)	−171.7(5)	−0.9(9)	−170.9(6)	–	12.6(7)	47.4(7)
21 ^(g)^	604432	[44]	*R*	Secondary amine	Secondary alkyl group	Primary alkyl group	−4.1(5)	−176.3(3)	8.7(5)	−170.3(3)	17.1	15.3(4)	51.7(4)
22 ^(g)^	605818	[45]	*R*	Secondary amine	Secondary alkyl group	Primary alkyl group	−15.6(2)	−174.1(1)	5.8(2)	−164.3(1)	−58.3	6.9(2)	52.1(1)
23	638938	[46]	*R*	Secondary amine	Primary alkyl group	Primary alkyl group	−6.2(5)	−173.8(3)	−1.8(5)	−173.7(3)	–	27.7(4)	51.2(4)
24 ^(g)^	675390	[47]	*R*	Secondary amine	Secondary alkyl group	Primary alkyl group	−14.6(2)	−174.7(1)	10.1(3)	−164.8(2)	−56.5	9.2(2)	50.4(2)
25 ^(g)^	706349	[48]	*R*	Secondary amine	Secondary alkyl group	Primary alkyl group	−13.7(4)	−172.3(3)	5.9(5)	−168.6(3)	19(2)	19.3(4)	59.4(4)
26 ^(h)^	707825	[49]	*S*	Secondary amine	Me	Secondary alkyl group	8.6(3)	173.1(2)	0.1(3)	173.6(2)	–	−12.7(3)	−50.0(3)
27 ^(h)^	707825	[49]	*S*	Secondary amine	Me	Secondary alkyl group	4.0(3)	176.7(2)	0.3(3)	−174.7(2)	–	−14.1(3)	−51.0(2)
28 ^(h)^	707825	[49]	*S*	Secondary amine	Me	Secondary alkyl group	1.1(3)	178.0(2)	1.3(3)	−176.7(2)	–	–18.4(3)	−48.3(3)
29 ^(h)^	707825	[49]	*S*	Secondary amine	Me	Secondary alkyl group	3.6(3)	177.1(2)	1.2(3)	–179.7(2)	–	−9.4(3)	−46.0(3)
30	766837	[50]	*S*	Secondary amine	Primary alkyl group	Primary alkyl group	9.4(2)	175.1(1)	2.0(2)	177.8(1)	–	−14.7(2)	−44.7(1)
31 ^(g)^	830079	[51]	*R*	Secondary amine	Tertiary alkyl group	Primary alkyl group	−18(1)	−173.1(6)	−1(1)	−178.3(7)	–	20(1)	55.8(9)
32	1104875	[52]	*R*	Secondary amine	Primary alkyl group	Primary alkyl group	−2.2(2)	−176.5(2)	−5.2(3)	−176.7(2)	–	27.1(2)	45.2(2)
33	1105464	[53]	*R*	Secondary amine	Primary alkyl group	Primary alkyl group	−8.2(8)	178.4(5)	−0.7(9)	−172.4(6)	–	12.0(8)	46.6(8)
34	1105464	[53]	*R*	Secondary amine	Primary alkyl group	Primary alkyl group	–9.0(8)	–175.6(5)	–6.1(9)	–168.0(6)	–	13.7(8)	45.8(7)
35 ^(g)^	1267150	[54]	*S*	Secondary amine	Secondary alkyl group	Primary alkyl group	7.1(6)	176.2(4)	−3.0(7)	−179.1(4)	19.5	−15.3(6)	−42.6(5)
36 ^(g)^	1267151	[54]	*R*	Secondary amine	Secondary alkyl group	Primary alkyl group	−14.0(7)	−170.3(4)	8.4(7)	−163.3(5)	24.4	29.7(7)	59.7(6)
37 ^(g)^	1280861	[55]	*R*	Secondary amine	Secondary alkyl group	Primary alkyl group	−15.6(6)	−170.9(4)	11.4(7)	−174.1(4)	−15.1	28.5(7)	50.1(6)
38 ^(g)^	1280861	[55]	*R*	Secondary amine	Secondary alkyl group	Primary alkyl group	−5.5(6)	−177.6(4)	7.8(7)	−177.4(4)	−17.0	25.0(6)	41.3(6)
39	1294281	[56]	*R*	Secondary amine	Primary alkyl group	Primary alkyl group	−6(1)	−175.0(7)	1(1)	−172.0(7)	–	26(1)	44.0(9)
40	1294281	[56]	*R*	Secondary amine	Primary alkyl group	Primary alkyl group	−15(1)	−172.9(7)	1(1)	−163.7(8)	–	14(1)	47(1)
41 ^(i)^	113953	[57]	*R*	Aniline derivative	*ortho*-Substituted phenyl group	H	−17(1)	−174.9(6)	−11(1)	178.6	–	8(1)	54.6(8)
42 ^(i)^	113953	[57]	*R*	Aniline derivative	*ortho*-Substituted phenyl group	H	−28(1)	−168.0(7)	−1(1)	−170.3	–	47.7(9)	65.9(8)
43 ^(f,i)^	113953	[57]	*R*	Aniline derivative	*ortho*-Substituted phenyl group	H	−58.4(9) ^(e)^	−175.1(6)	−1(1)	176.4	–	−56.4(9)	−153.6(6)
44 ^(i)^	113953	[57]	*R*	Aniline derivative	*ortho*-Substituted phenyl group	H	−15(1)	−170.8(6)	6(1)	175.4	–	36.1(9)	57.7(8)
45 ^(i)^	1310848	[58]	*R*	Aniline derivative	*ortho*-Substituted phenyl group	H	−2(2)	−172(1)	−5(3)	–	–	35(2)	51(2)
46 ^(f,i)^	1310848	[58]	*R*	Aniline derivative	*ortho*-Substituted phenyl group	H	−60(2) ^(e)^	179(2)	3(3)	–	–	−34(2)	−172(2)
47 ^(i)^	1310848	[58]	*R*	Aniline derivative	*ortho*-Substituted phenyl group	H	−41(2) ^(e)^	−169(2)	0(3)	–	–	13(2)	71(2)
48 ^(f,i)^	1310848	[58]	*R*	Aniline derivative	*ortho*-Substituted phenyl group	H	−51(2) ^(e)^	−165(2)	−1(3)	–	–	85(2)	−154(2)
49	655554	[59]	*R*	Benzotriazole	*ortho*-Substituted phenyl group	N	−9.3(1)	−175.21(7)	−2.8(1)	178.81(8)	–	25.7(1)	46.6(1)
50 ^(f)^	1142231	[60]	*S*	Diethyl 1-aminoalkylphos-phonate derivative	1-(Diethoxy-phophoryl)alkyl group	H	57(3) ^(e)^	169(2)	9(4)	–	−14	77(2)	148(2)
51	1142231	[60]	*S*	Diethyl 1-aminoalkylphos-phonate derivative	1-(Diethoxy-phophoryl)alkyl group	H	24(3)	172(2)	4(3)	–	−12	−35(2)	−65(2)
52	1236701	[61]	*R*	Diethyl 1-aminoalkylphos-phonate derivative	1-(Diethoxy-phophoryl)alkyl group	H	−23.1(5)	−171.5(3)	−3.1(6)	176.6	19.2	7.3(5)	59.3(4)
53	1236702	[61]	*R*	Diethyl 1-aminoalkylphos-phonate derivative	1-(Diethoxy-phophoryl)alkyl group	H	−19.4(6)	−174.3(4)	−3.2(7)	177.2	18.8	5.8(6)	55.8(5)
54	1236703	[61]	*R*	Diethyl 1-aminoalkylphos-phonate derivative	1-(Diethoxy-phophoryl)alkyl group	H	−24(2)	−166(1)	4(2)	−173	11	31(2)	59(2)
55	1236703	[61]	*R*	Diethyl 1-aminoalkylphos-phonate derivative	1-(Diethoxy-phophoryl)alkyl group	H	−24(2)	−166(1)	9(2)	−171	−14	35(2)	57(1)
56	1216345	[62]	*R*	Oxazolidine-2-selone derivative	Selenoxo group	Secondary alkyl group	−15.9(4)	−172.9(2)	24.5(4)	−147.8(3)	–	18.5(4)	56.6(3)
57	630372	[63]	*R*	Thiocarbamide derivative	*N*-Substituted thiocarbamoyl group	Secondary alkyl group	−16.7(3)	−174.1(2)	11.2(4)	−152.6(2)	–	24.8(3)	57.7(3)
58	143886	[64]	*R*	*p*-Toluene-sulfonamide derivative	*p*-Toluene-sulfonyl group	Primary alkyl group	−5.4(6)	−177.4(3)	7.4(5) ^(j)^	−177.6(4)	–	18.2(6)	37.8(5)

^(a)^ The original dihedral angles were cited for both (*R*_acid_)- and (*S*_acid_)-MTPA amides. In the following sections, (*S*_acid_)-MTPA amides were processed as (*R*_acid_)-MTPA amides with plus/minus sign reversal. ^(b)^ The same CCDC numbers stand for multiple conformers in a lattice or bis- and tetrakis-MTPA amides. ^(c)^ X1′′ stands for the atom at the α-position of substituent R*^ap^* in the amine moiety. ^(d)^ Mosher‑type MTPA amides prepared from primary amines possessing secondary alkyl groups. ^(e)^ These ten entries were omitted from the calculation of the average dihedral angle *θ*^1^. ^(f)^ The minor conformer in which the amide carbonyl group and the methoxy group were *anti*. ^(g)^ The tertiary amides exhibiting *Z* forms (R*^sp^* > R*^ap^*). All precursors of these *Z* amides were the cyclic secondary amines. We refer to *Z* and *E* as the forms in which the larger *N*-substituent is *sp* and *ap* to the carbonyl oxygen atom, respectively [72]. ^(h)^ The four conformers of CCDC 707825 (*N*-methylamide derivative) only exhibited *E* forms (R*^sp^* < R*^ap^*) in the amide moieties [49]. ^(i)^ The tetrakis-MTPA amides of ruthenium(II) porphyrin complexes. ^(j)^ The dihedral angle of S–N–C1–O1 was cited.

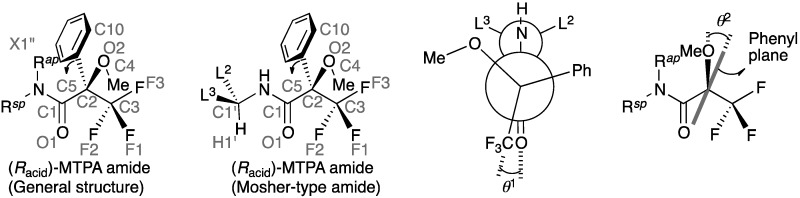



All obtained MTPA amide moieties (a total of 58) were processed as *R*_acid_-enantiomers in the following sections; that is, the dihedral angles of (*S*_acid_)-MTPA amides cited in Table 1 were substituted by those of the mirror images (*i.e.*, *R*_acid_-enantiomers) with plus/minus sign reversal. Despite the various structures of the amine moiety, the MTPA amide moieties showed little conformational diversity. Therefore, the conformations of all MTPA amide moieties were processed together, excluding the dihedral angle H1′–C1′–N–C1, which was specific to the MTPA amides prepared from amines possessing a secondary alkyl group (see Section 2.6).

### 2.2. Dihedral Angles of Amide Carbonyl Group and Trifluoromethyl Group: O1–C1–C2–C3

**Figure 3:**
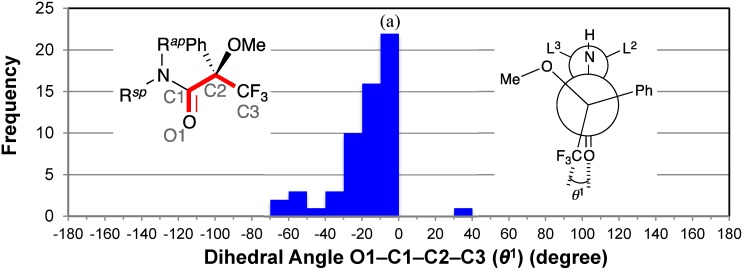
Histogram for dihedral angles between the amide carbonyl and trifluoromethyl groups of all MTPA amides. ^(a)^ The bar indicates the frequency of −10 < *θ*^1^ ≤ 0; and the same is true for the others.

The distribution of the dihedral angles O1–C1–C2–C3 (*θ*^1^) exhibited a concentration of entries between −30° and 0° (Figure 3). All dihedral angles ranged from −70° to +40°; the median angle was −14.5°. These data confirmed Mosher’s hypothesis that the carbonyl and trifluoromethyl groups of the MTPA amide are *syn* [3]. In addition, the majority of dihedral angles *θ*^1^ (48/58, *ca.* 83%) ranged from −30° to 0° with the average angle *θ*^1^ of −13°. That is, the carbonyl group was close to the phenyl group. This phenomenon was also observed for the MTPA esters and MTPA salt; we observed the short contacts between the two oxygen atoms and the *ortho‑*hydrogen atoms of phenyl group in the MTPA anion [9].

All the outliers were the dihedral angles *θ*^1^ of the secondary amides. An irregular *θ*^1^ of +30.3(3)° represents CCDC 222942 [28] in which the intramolecular aromatic C–H···π interactions and N–H···O hydrogen bond were observed.

The five entries around −60° were indicative of a minor conformer in which the amide carbonyl group and the methoxy group were *anti* [*i.e*., CCDC 703912 [32], −67.7 (6)°; CCDC 1142231 [60], −57(3)° as *R*_acid_-enantiomer; CCDC 113953 [57], −58.4(9)°; CCDC 1310848 [58], −60(2)° and −51(2)°] (see Section 2.7 and Section 2.8). The amine moiety of CCDC 703912 has a linear conjugated diene structure. In the case of CCDC 1142231, the phenyl group of the MTPA moiety formed an intramolecular C–H···O hydrogen bond [73] with the oxygen atom of the amine moiety. The other minor *anti* conformers (*i.e.*, CCDC 113953 and CCDC 1310848) were the tetrakis-MTPA amide derivatives of ruthenium(II) porphyrin complexes.

The tetrakis-MTPA amide derivative CCDC 1310848 also exhibited another irregular *θ*^1^ of −41(2)°. CCDC 739753 [34] showed an irregular *θ*^1^ of −36.0(2)°; the methoxy group of the MTPA moiety formed an intramolecular aromatic C–H···π interaction with the phenyl group of the amine moiety. CCDC 122980 [36] also exhibited an irregular *θ*^1^ of −31(1)°; the methoxy group of the MTPA moiety formed an intramolecular C–H···O hydrogen bond with the oxygen atom of the amine moiety. In addition, CCDC 603055 [29] showed an irregular *θ*^1^ of −35.0(4)° as *R*_acid_-enantiomer; the amine moiety possessed a sulfonamide moiety and a phenyl group.

Previously, the dihedral angle *θ*^1^ was discussed in relation to the steric repulsion between the phenyl group and L^2^ or L^3^ substituents of the amine moiety; it was estimated that the larger repulsion resulted in a lager dihedral angle *θ*^1^, and a smaller deshielding of the fluorine atoms by the amide or ester carbonyl groups [4]. However, Kusumi *et al.* observed the inaccuracies of the MTPA method using ^19^F-NMR spectroscopy [5,8]. In 2007, Brand *et al*. reported that the origin of the *sp* conformation is the hyperconjugative interactions between the carbonyl group and the electronegative trifluoromethyl group [74].

### 2.3. Staggered Conformation of Trifluoromethyl Group: C1–C2–C3–F3

**Figure 4:**
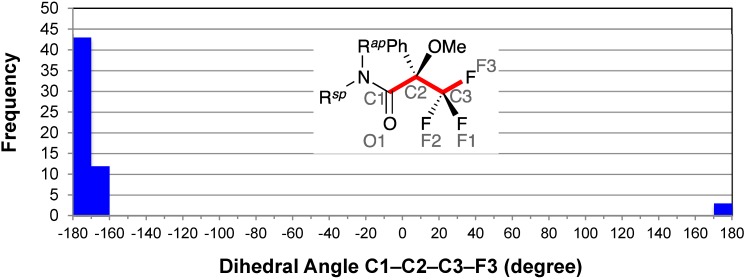
Histogram for dihedral angles C1–C2–C3–F3 of all MTPA amides. F3 is the fluorine atom *ap* to C1. The possible range of the dihedral angle C1–C2–C3–F3 is from −180° to −120° and from +120° to +180°.

The distribution of the dihedral angle C1–C2–C3–F3 showed a concentration of entries around −180° (Figure 4). The median angle was −174°. This was suggestive of the staggered conformation of the trifluoromethyl group [9]; that is, the two oxygen atoms O1 and O2 were as far as possible from the fluorine atoms.

Khan *et al*. reported the nonequivalence of three fluorine atoms of the MTPA amide prepared from secondary amines on the ^19^F-NMR spectra at low temperatures [75]. They also reported that barriers to the hindered rotation on the C2–C3 bonds were in the range of 36–46 kJ/mol. This suggested the severe steric crowding of the MTPA moiety.

### 2.4. Resonance Effects of Amide Bond: C1′–N–C1–O1

**Figure 5:**
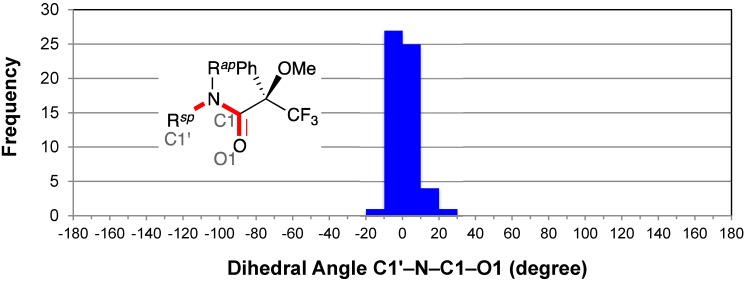
Histogram for the dihedral angles C1′–N–C1–O1 of all MTPA amides. The α-carbon atom of substituent R*^sp^*, which is *sp* to the amide carbonyl group, was defined as C1′. The possible range of the dihedral angle C1′–N–C1–O1 is from −90° to +90°.

**Figure 6:**
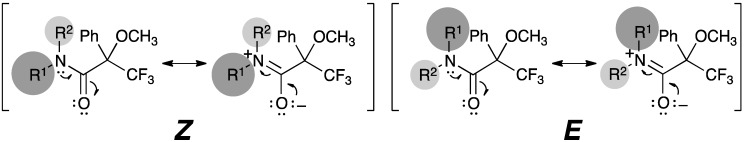
*Z*/*E* forms and their resonance hybrids of MTPA amides. R^1^ indicates the substituents with higher Cahn–Ingold–Prelog (CIP) priority (e.g., secondary alkyl group); R^2^ indicates the substituents with lower CIP priority (e.g., hydrogen atom and methyl group).

The distribution of the dihedral angle C1′–N–C1–O1 exhibited a concentration of entries around 0° (Figure 5). The average angle and the median angle were +2° and +0.8°, respectively. This represents the resonance effects of the amide bond (Figure 6). Similar planarity was observed in the ester moieties of the crystalline MTPA esters [9].

The trivalent nitrogen atom afforded the Z and *E* forms in the amide moiety (Figure 6). All crystalline secondary MTPA amides prepared from primary amines exhibited the *Z* form, in which the *N*-substituent was *sp* to the amide carbonyl group (Figure 2 and Table 1).

In the case of tertiary MTPA amide moieties prepared from secondary amines, the ratio of *Z*/*E* forms was 13:4 (Table 1). The MTPA amide moieties prepared from cyclic secondary amines exhibited the *Z* forms (*i.e.*, CCDC 140352 [38], CCDC 167289 [39], CCDC 241708 [40], CCDC 251663 [41], CCDC 604432 [44], CCDC 605818 [45], CCDC 675390 [47], CCDC 706349 [48], CCDC 830079 [51], CCDC 1267150 [54], CCDC 1267151 [54], and the two conformers of CCDC 1280861 [55]).

The four conformers of an *N*-methyl MTPA amide exhibited the *E* forms (*i.e.*, CCDC 707825 [49]). It is noteworthy that the two aromatic rings of the larger secondary alkyl group R^1^ were bound to the MTPA’s phenyl groups via the aromatic C–H···π and π···π interactions [73,76], respectively. By contrast, Nakagawa and Somei reported the *Z* form of a crystalline *N*-methyl MTPA amide in the course of the total synthesis of ergot alkaloids [65].

It is possible that the application of Mosher’s method using ^1^H-NMR could be expanded to the cyclic secondary amines [77,78]. In 1996, Hoye and Renner applied the MTPA method for assignment of the absolute configuration in chiral cyclic amines; they observed equilibrium mixtures of the *Z* and *E* forms of amide moieties in the ^1^H-NMR spectra [77]. Similar peptidyl-prolyl isomerization is a key issue in protein chemistry [79]. In 2001, Azumaya reported the *E*‑preference of aromatic *N*-methylamides (e.g., *N*-methylbenzanilide) [80].

### 2.5. Resonance Effects of Amide Bond: X1′′–N–C1–O1

**Figure 7:**
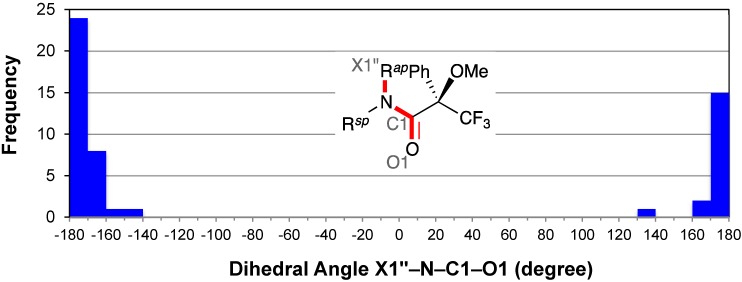
Histogram for dihedral angles X1′′–N–C1–O1 of all MTPA amides. X1′′ is the α-atom of R*^ap^*, which is *ap* to the amide carbonyl group. The possible range of the dihedral angle X1′′–N–C1–O1 is from −180° to −90° and from +90° to +180°.

The distribution of the dihedral angle X1′′–N–C1–O1 also exhibited a concentration of entries around −180° (Figure 7). The median angle was −174°. This also represents for the resonance effects of the amide bond (see above).

### 2.6. Conformation of the Amine Moiety: H1′–C1′–N–C1

**Figure 8:**
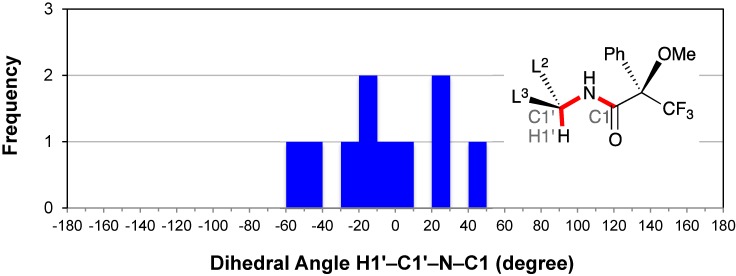
Histogram for dihedral angles H1′–C1′–N–C1 of the MTPA amides prepared from primary amines possessing a secondary alkyl group.

The distribution of the dihedral angle H1′–C1′–N–C1 was examined in the case of the ten crystalline MTPA amides prepared from the primary amines possessing secondary alkyl groups (*i.e.*, Mosher‑type MTPA amides). All dihedral angles were distributed between −60° and +50° with an average angle of −5° (Figure 8). Besides, the median angle was −11°. These data agree with Mosher’s hypothesis of the MTPA plane [3,5]. In addition, the broad distribution of dihedral angles was indicative of a moderate conformational flexibility of the C1′–N bond. The same is true for the C1′–O bond of the crystalline MTPA esters prepared from secondary alcohols [9].

Rzepa analyzed the relationship between the dihedral angle H–N–C=O and the distance from H1’ of the amine moiety to the carbonyl oxygen atom in the crystalline secondary amides (a total of 619); that is, the major conformer exhibited a *syn*-co-planar alignment of the C–H bond with the plane of the C=O bond in the *Z* form [81].

### 2.7. Dihedral Angle between the Methoxy Group and Phenyl Group: O2–C2–C5–C10

**Figure 9:**
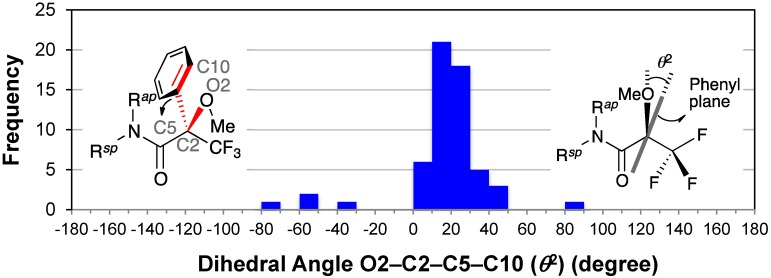
Histogram for dihedral angles O2–C2–C5–C10 (*θ*^2^) of all MTPA amides. C10 is the *ortho*‑carbon atom that provides a smaller absolute value. The possible range of the dihedral angle O2–C2–C5–C10 (*θ*^2^) is from −90° to +90°.

The distribution of the dihedral angles O2–C2–C5–C10 (*θ*^2^) exhibited a concentration of entries around +20° (Figure 9). The majority of dihedral angles (53/58, *ca.* 91%) ranged from 0° to +50°; the average angle of these major 53 conformers was +21°. Besides, the median angle for all 58 conformers was +20°.

These data confirmed Mosher’s hypothesis of MTPA amide, in which the (*R*_acid_)-MTPA’s phenyl group shields the amine’s substituent L^2^ (Figure 2a) [3]. Figure 9 also suggested that the substituent L^2^ is not just above the phenyl ring. We reported that the phenyl group was inclined by +19° in the MTPA ester [9].

The other five entries exhibited the minor conformer in which the amide carbonyl group and the methoxy group were *anti* (see Section 2.2 and Section 2.8). The crowded tetrakis-MTPA amides of ruthenium(II) porphyrin complexes (CCDC 113953 [57] and CCDC 1310848 [58]) exhibited the irregular dihedral angles *θ*^2^ [*i.e.*, −56.4(9)°, −34(2)°, and +85(2)°]. One of the conformer of CCDC 1142231 [60], in which the MTPA’s phenyl group forms the C–H···O hydrogen bond with the ethoxy oxygen atom of amine moiety, also exhibited an irregular dihedral angle *θ*^2^ [*i.e.*, −77(2)° as *R*_acid_-enantiomer]. The rest was CCDC 703912 [32] [*i.e.*, −58.9(7)°], which contains the linear conjugated diene in the amine moiety.

The dihedral angle *θ*^2^ has a significant influence on shielding by the phenyl group according to ^1^H-NMR spectroscopy. The dihedral angle *θ*^2^ also influenced the crystallization, because the intramolecular interactions (e.g., C–H···π and π···π interactions) are crucial for chiral recognition by the resolving agents [71].

### 2.8. Conformation of the Methoxy Group: C1–C2–O2–C4

**Figure 10:**
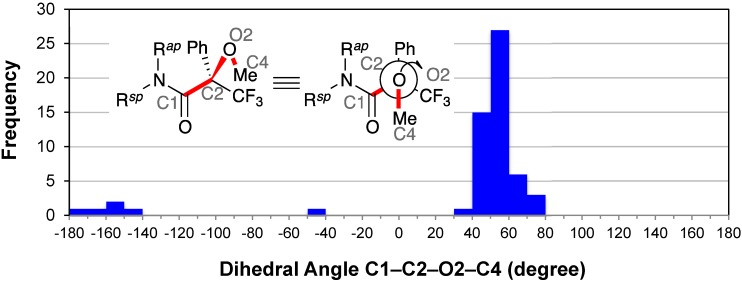
Histogram for dihedral angles between the methoxy and phenyl groups of all MTPA amides.

The distribution of the dihedral angles C1–C2–O2–C4 exhibited a concentration of entries around +50° (Figure 10). The majority of dihedral angles (52/58, *ca*. 90%) ranged between +30° and +80°; the average dihedral angle for the major 52 conformers was +54°. Besides, the median angle for all 58 conformers was +51°. That is, the methyl group of the methoxy moiety was *ap* to the phenyl group. This extended form was also observed as the major conformer in the MTPA esters, along with the other minor conformers [9]. Khan *et al*. reported that *θ*^2^ was relaxed to +50° using the MNDO method in the case of (*R*)-*N*,*N*′-dimethyl-3,3,3-trifluoro-2-methoxy-2-phenylpropanamide [75].

An irregular dihedral angle −44.4(4)° was observed in the conformer of CCDC 222942 [28], in which the phenyl group of the amine moiety formed the C–H···π interactions with the MTPA’s methoxy moiety (see Section 2.2.). Similar C–H···π interactions were observed in CCDC 739753 [34] and CCDC 1104875 [52]. It is possible that the methoxy moiety could interact with the amine moieties through weak intramolecular interactions (e.g., C–H···π interaction and C–H···O hydrogen bond) [9,73,76].

The tetrakis-MTPA amides of ruthenium(II) porphyrin complexes again yielded the irregular dihedral angles (*i.e.*, CCDC 113953 [57], −153.6(6)°; CCDC 1310848 [58], −172(2)°, and −154(2)°). The MTPA amide of CCDC 703912 [32], which has a linear conjugated diene moiety in the amine’s substituent L^2^, exhibited another irregular dihedral angle of −162.7(5)°. One of the conformers of CCDC 1142231 [60] also exhibited the irregular dihedral angle (*i.e*., −148(2)° as *R*_acid_-enantiomer); these represent the minor conformer in which the amide carbonyl group and the methoxy group were *anti*.

It is noteworthy that Saigo reported that agreement of the molecular lengths is important for the successful resolution via the diastereomeric salt formation method [71]. We reported that the agreement of the molecular lengths of acid/alcohol moieties is important for the crystallization of MαNP esters [16].

The crystal structures of CCDC 199868 [27] and CCDC 678252 [31] contain the typical conformations of Mosher-type MTPA amides.

## 3. Experimental Section

### 3.1. Database Study of MTPA Amide

The accessible 41 crystal structures of MTPA amides reported from 1985 to 2011 [27,28,29,30,31,32,33,34,35,36,37,38,39,40,41,42,43,44,45,46,47,48,49,50,51,52,53,54,55,56,57,58,59,60,61,62,63,64,65,66] were examined. The original dihedral angles of (*R*_acid_)- and (*S*_acid_)-MTPA amides were shown in Table 1. In this study, all MTPA amides were processed as (*R*_acid_)-MTPA amides; that is, the dihedral angles of (*S*_acid_)-MTPA amides cited in Table 1 were substituted by those of the mirror images (*i.e.*, *R*_acid_-enantiomers) with plus/minus sign reversal [82]. Each of bis- and tetrakis-MTPA amides (*i.e.*, CCDC 1280861 [55], CCDC 113953 [57], and CCDC 1310848 [58]), as well as the different conformers in a lattice (*i.e.*, CCDC 199868 [27], CCDC 651954 [30], CCDC 678252 [31], CCDC 707825 [49], CCDC 1105464 [53], CCDC 1142231 [60], CCDC 1236703 [61], and CCDC 1294281 [56]), was processed individually. This procedure yielded 58 of (*R*_acid_)-MTPA amide moieties. These MTPA amide moieties were prepared from various amine moieties; that is, primary amines (a subtotal of 14), secondary amines (26), aniline derivatives [tetrakis-MTPA amides of ruthenium(II) porphyrin complexes] (8), benzotriazole (1), diethyl 1-aminoalkylphosphonate derivatives (6), an oxazolidine-2-selone derivative (1), a thiocarbamide derivative (1), and a *p*-toluenesulfonamide derivative (1). The dihedral angles were obtained using Mercury software (Ver. 3.5.1) [83] from the CIF files. The CIF files listed in Table 1 contain the supplementary crystallographic data for this paper. These data can be obtained free of charge via http://www.ccdc.cam.ac.uk/conts/retrieving.html (or from the CCDC, 12 Union Road, Cambridge CB2 1EZ, UK; Fax: +44 1223 336033; E-mail: deposit@ccdc.cam.ac.uk).

### 3.2. Caution

Acylation of an amine with (*S*)-MTPA chloride yields (*R*_acid_)-MTPA amide. In the same way, acylation of an amine with (*R*)-MTPA chloride yields (*S*_acid_)-MTPA amide. This nominal change in the absolute configuration has often caused confusion. The same is true for MTPA esters.

## 4. Conclusions

We conducted a database study of the crystal structures of MTPA amides deposited in the CSD. The properties of the major conformation of the MTPA amide elucidated from our database study confirmed Mosher’s empirical model on the conformation of MTPA amide; that is, the methine group of the amine moiety, the amide carbonyl group, and the trifluoromethyl group are on the MTPA plane. All secondary MTPA amides prepared from the primary amines exhibited the *Z* form in the amide moiety. The ratio of *Z*/*E* forms of amide moieties was 13:4 in the case of the tertiary MTPA amides prepared from the secondary amines; the cyclic secondary amines yielded the *Z* forms, whereas the *N*-methyl amines yielded the both *Z* and *E* forms. The amide carbonyl group was *sp* to the trifluoromethyl group with the average dihedral angle *θ*^1^ of −13°. The trifluoromethyl group was in the staggered conformation. In addition, the C1′–N bond of the amine moiety exhibited moderate conformational flexibility. The phenyl plane was inclined by *θ*^2^ = +21° from the O–C_chiral_ bond of the methoxy moiety. This dihedral angle *θ*^2^ was suggestive of the inefficient shielding of the phenyl ring. Finally, the methyl group of the methoxy moiety was *ap* to the *ipso*‑carbon atom of the phenyl group. These conformational properties were similar to those of the crystalline MTPA ester. Besides, the minor conformer of the crystalline MTPA amides was observed in which the amide carbonyl group and the methoxy group were *anti*. Mosher’s method using NMR spectroscopy is crucial for the structural elucidation of chiral amines in combination with X-ray crystallography. This report increases our understanding of Mosher’s method and acid **1** and can be used for crystal engineering of MTPA derivatives.
